# A Carrier of Disease Germs

**Published:** 1913-01

**Authors:** 


### A Carrier of Disease Germs.

Springfield, Mass., had the highest mortality rate from infantile paralysis two years ago of any city in the country, it is said. Last week it was found that cats there were infected with the disease and some of them have been sent to Boston for an investigation as to whether the malady may not be due to them.

In referring to the matter, the *Scranton Tribune-Republican* says: "If it is demonstrated that infantile paralysis has been distributed by the house cat, another evil will be placed at the door of the animal that is supposed to be active in disseminating tuberculosis, diphtheria and many other infectious complaints. If the cat is really responsible for infantile paralysis, it will also explain the origin of many mysterious cases that have heretofore puzzled the health officials."

It seems to be generally agreed that cats transmit the germs of various diseases, among them those mentioned above. It is therefore probable that it also carries those of infantile paralysis. The animal is more or less a danger to children.—*Bulletin S. B. H., Texas.*

